# Rural–urban differences in secular trends of locoregional treatment for ductal carcinoma in situ: A patterns of care analysis

**DOI:** 10.1002/cam4.4605

**Published:** 2022-02-11

**Authors:** Danielle Riley, Elizabeth A. Chrischilles, Ingrid M. Lizarraga, Mary Charlton, Brian J. Smith, Charles F. Lynch

**Affiliations:** ^1^ Department of Epidemiology, College of Public Health University of Iowa Iowa City Iowa USA; ^2^ Department of Surgery University of Iowa Hospitals and Clinics Iowa City Iowa USA; ^3^ Holden Comprehensive Cancer Center University of Iowa Iowa USA

**Keywords:** breast cancer, breast‐conserving surgery, ductal carcinoma in situ, mastectomy, post‐operative radiation therapy, quality of care, rural

## Abstract

**Precis:**

Omission of PORT following BCS remains high among rural patients despite evidence that PORT leads to a significant reduction in the risk of local recurrence. Further research is needed to examine the impact of rural residence on treatment choices and develop methods to ensure equitable care among all breast cancer patients.

**Background:**

Despite national guidelines, debate exists among clinicians regarding the optimal approach to treatment for patients diagnosed with ductal carcinoma in situ (DCIS). While regional variation in practice patterns has been well documented, population‐based information on rural–urban treatment differences is lacking.

**Methods:**

Data from the SEER Patterns of Care studies were used to identify women diagnosed with histologically confirmed DCIS who underwent cancer‐directed surgery in the years 1991, 1995, 2000, 2005, 2010, and 2015. Adjusted odds ratios (aORs) and 95% confidence intervals (CIs) were estimated using weighted multivariable logistic regression to evaluate cancer‐directed surgery and use of post‐operative radiation therapy (PORT).

**Results:**

Of the 3337 patients who met inclusion criteria, 27% underwent mastectomy, 26% underwent breast‐conserving surgery (BCS) without PORT, and 47% underwent BCS with PORT. After adjustment for other covariates, there was no difference in the likelihood of receiving mastectomy between rural and urban patients (aOR = 0.65; 95% CI 0.37–1.14). However, rural residents were more likely than urban residents to have mastectomy during 1991/1995 (aOR = 1.78; 95% CI 1.09–2.91; *p*_interaction_ = 0.022). Across all diagnosis years, patients residing in rural areas were less likely to receive PORT following BCS compared to urban patients (aOR = 0.35; 95% CI 0.18–0.67).

**Conclusions:**

Omission of PORT following BCS remains high among rural patients despite evidence that PORT leads to a significant reduction in the risk of local recurrence. Further research is needed to examine the impact of rural residence on treatment choices and develop methods to ensure equitable care among all breast cancer patients.

## Graphical abstract

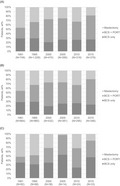

## 1 INTRODUCTION

Ductal carcinoma in situ (DCIS) is a noninvasive tumor characterized by the presence of abnormal cells confined to the breast ducts.^1^ Approximately 18% of all breast cancers diagnosed among women in the United States (US) are DCIS, with an estimated 50,000 new cases diagnosed annually.^2^ DCIS is most commonly identified as microcalcifications on mammography with fewer than 10% of cases presenting with a palpable mass.^3^ Studies suggest that 36% of DCIS cases will progress to an invasive cancer over a period of 10 or more years if left untreated.^4^

For this reason, treatment of DCIS is routinely recommended with the goal of minimizing recurrence of in situ disease or the development of invasive breast cancer. Current clinical practice guidelines set by the National Comprehensive Cancer Network (NCCN) recommend either total mastectomy (with or without sentinel lymph node biopsy [SLNB]) or breast‐conserving surgery (BCS) as primary treatment options. For patients who undergo BCS and are considered “high‐risk” for recurrence (i.e., large tumor size, higher tumor grade, palpable mass, positive surgical margins, and younger age at diagnosis), post‐operative radiation therapy (PORT) should be administered.^5^ However, because the natural history of DCIS continues to be poorly understood,^6^ there remains considerable debate among clinicians about the optimal treatment approach resulting in observations of persistent and substantial variation in clinical practice patterns across the US.^7^, ^8^, ^9^, ^10^, ^11^, ^12^, ^13^

Previous studies have reported significant differences in locoregional treatment patterns among patients diagnosed with invasive breast cancer by geographic region and across the rural–urban continuum.^14^, ^15^, ^16^, ^17^, ^18^, ^19^ However, few studies have evaluated whether such differences also exist among DCIS patients residing in rural versus urban areas.^12^, ^20^, ^21^, ^22^ The purpose of this study was compare the type of cancer‐directed surgery performed and use of PORT between rural and urban women diagnosed with DCIS between 1991 and 2015.

## 2 METHODS

We used data from the National Cancer Institute's (NCI) SEER Patterns of Care (POC) studies. SEER registries cover approximately 35% of the U.S. population and routinely collect patient demographic and primary tumor characteristics as well as the first course of treatment and vital status for every cancer case diagnosed within 19 geographic regions.^23^ To obtain more detailed treatment information including the use of radiation therapy that may be administered in the outpatient setting, annual POC studies are conducted by the NCI on a sample of patients with select cancers. In situ breast cancer, including DCIS, was selected as a POC site in 1991, 1995, 2000, 2005, 2010, and 2015.^24^

Each POC dataset included a random sample of in situ cases selected within each registry stratified by race/ethnicity and age (≤50, >50). Racial/ethnic groups other than non‐Hispanic whites were oversampled to provide more stable population estimates. Trained abstractors collected all treatment information from the medical record up to 2 years post‐diagnosis. As a measure of quality control, a blind re‐abstraction of the medical record was performed on a random 5% sample of cases within each registry. In addition, each treating physician or physician's office was mailed a form to verify all abstracted treatments and report any additional therapies not already included on the abstraction form.^25^ Verification of therapies were obtained for 81% of patients included in the analysis.

The University of Iowa Institutional Review Board determined that this study did not meet the criteria for human subjects' research and was exempt from further review.

## 3 STUDY POPULATION

Our study population included 3826 women aged 20 years and older with newly diagnosed DCIS who underwent cancer‐directed surgery during the years of 1991, 1995, 2000, 2005, 2010, and 2015. Patients were excluded from each POC study if they had a previous cancer diagnosis, except for non‐melanoma skin cancer, were diagnosed at autopsy or on the death certificate, or were diagnosed with a simultaneous primary cancer. For this analysis, we also excluded patients with non‐DCIS histology codes (8000, 8010, 8050, 8140, 8210, 8520, 8540, 8543, 8573) (*n* = 470) as well as those coded with non‐in situ staged tumors (*n* = 19). Our final sample included 3337 women diagnosed with pure DCIS at final pathology.

## 4 MEASURES

### 4.1 Defining locoregional treatment

Patients were categorized based on the type of cancer‐directed surgery performed as part of the first‐course therapy using SEER site‐specific surgery codes. BCS was defined using codes for partial mastectomy with or without nipple resection, lumpectomy, excisional biopsy, re‐excision of biopsy site, wedge resection, quadrantectomy, segmental mastectomy, or tylectomy. Mastectomy was defined using surgery codes for subcutaneous mastectomy, total (simple) mastectomy, modified radical mastectomy, radical mastectomy, or extended radical mastectomy. Due to the small number of bilateral mastectomies, unilateral and bilateral mastectomies were combined. PORT was determined from the administration sequence of radiation with surgery.

### 4.2 Defining rurality

The primary exposure of interest was rural–urban residence. We used rural–urban continuum codes (RUCC) to classify patients' level of rurality based on the ZIP code of residence at time of diagnosis. RUCCs refer to a county‐based classification scheme maintained by the United States Department of Agriculture.^26^ Patients diagnosed in 1991 or 1995 were linked to 1993 RUCC codes, those diagnosed in 2000 or 2005 to 2003 RUCC codes, and those diagnosed in 2010 and 2015 to 2013 RUCC codes. We considered urban patients to be those residing in metropolitan areas (RUCC codes 1–3) and rural patients to be those residing in non‐metropolitan areas (RUCC codes 4–9).

### 4.3 Demographic and clinical characteristics

Patient‐level demographics included age at diagnosis, race/ethnicity, marital status, region of residence and insurance status. Region of residence was categorized based on US Census Divisions as Northeast (Connecticut, New Jersey), Midwest (Detroit, Iowa), South (Atlanta, Kentucky, Louisiana), and West (San Francisco, Hawaii, New Mexico, Seattle, Utah, Alaska, San Jose/Monterey, Los Angeles, Greater California). Of note, the following registries did not contribute data to all six POC study years: Hawaii (1995), Utah (1995), Alaska (1991, 1995, 2005, 2010, 2015), San Jose/Monterey (1991, 1995), Los Angeles (1991), Greater California (1991, 1995, 2000), Kentucky (1991, 1995, 2000, 2005) Louisiana (1991, 1995, 2000) and New Jersey (1991, 1995, 2000). Insurance status was defined as insured (private, Medicare, CHAMPUS, Veterans Affairs, and other governmental insurance), Medicaid coverage (any Medicaid, including Indian Health Service) and uninsured. Patients with no known insurance coverage at the time of diagnosis were assigned to the uninsured category. Clinical characteristics included year of diagnosis, tumor size, tumor grade, margin status, estrogen‐receptor (ER) status, laterality, comorbidity status, and time to surgery. Comorbidity status at the time of treatment was calculated using the Charlson Comorbidity Index Score and dichotomized as 0 and ≥1.^27^ Time to surgery was calculated as the number of days between initial biopsy and cancer‐directed surgery.

### 4.4 Hospital characteristics

Characteristics of the hospital that administered the most definitive surgical treatment were provided by the American Hospital Association and included hospital bed size, hospital classification, and presence of an approved residency training program by the Accreditation Council for Graduate Medical Education. Hospital classification was categorized into two groups: public and private. Public hospitals included all nonfederal (city, county, state) and Federal (Air force, Army, Navy, Public Health Service, Veterans Administration, Public Health Service Indian Service, Department of Justice, other Federal facilities) facilities. All not‐for‐profit (church‐operated, other not‐for‐profit) and for‐profit (individual, partnership, corporation) facilities were categorized as private hospitals.

### 4.5 Statistical analysis

Rao‐Scott *χ* ^2^ tests were used to examine the unadjusted associations between categorical variables and rural–urban residence. Unadjusted weighted logistic regression was used to examine trends among ordinal variables. Weighted multivariable logistic regression was used to evaluate the association of rural–urban residence with type of cancer‐directed surgery and use of PORT, after controlling for other covariates. Probability sampling weights were used to account for the oversampling of nonwhite patients and to obtain representative estimates for all eligible patients in the study areas. The weights were calculated as the inverse of the sampling proportion for each sample stratum (defined by stage, race/ethnicity, SEER registry and year of diagnosis). Results of each model were presented as adjusted odds ratios (aORs) using a 95% confidence interval (CI). Categorical variables were reported as frequencies and weighted percentages. Multiplicative effect modification was explored using interaction terms between rural–urban residence and year of diagnosis as well as region of residence. We used SAS software, version 9.1.4 (SAS Institute Inc., Cary, North Carolina) for all analyses, and considered two‐sided *p*‐values < 0.05 to be statistically significant.

## 5 RESULTS

Of the 3337 patients who met inclusion criteria, approximately 9% (*n* = 259) were rural. The proportion of rural patients increased significantly over time (*p*_trend_ = 0.001) as the number of registries participating in the POC studies increased (Table 1). Rural patients were more likely to be non‐Hispanic white (*p* < 0.0001) and reside in states associated with Midwestern and Southern registries (*p* < 0.0001). Rural patients were more likely to experience longer delays between initial biopsy and cancer‐directed surgery (*p*_trend_ = 0.049). Compared to urban patients, rural patients were more likely to be treated with BCS only and less likely to undergo BCS with PORT or mastectomy. Compared to urban patients, a higher proportion of rural patients received definitive treatment at low‐volume (characterized by total hospital bed size) (*p*_trend_ = 0.001), public hospitals (*p* < 0.0001), with no known residency training programs (*p* < 0.0001). There were no clinically meaningful differences in rural–urban residence by tumor size, tumor grade, surgical margin status, ER status, or laterality.

**TABLE 1:** Characteristics of DCIS patients stratified by rural–urban residence

	Rural (*n* = 259)	Urban (*n* = 3078)	
	*N* (wt%^a^)	*N* (wt%^a^)	*p*‐value
Age at diagnosis, years			
<50	93 (19.9)	1285 (25.9)	0.491^b^
50–59	55 (33.8)	736 (26.2)	
60–69	56 (20.4)	591 (24.9)	
≥70	55 (25.9)	466 (23.0)	
Race/ethnicity			
Non‐Hispanic, White	202 (88.7)	1577 (66.9)	**<0.0001** ^c^
Non‐Hispanic, Black	9 (1.1)	651 (10.8)	
Other^d^/Unknown	48 (10.2)	850 (22.3)	
Marital status at diagnosis			
Married/Living with partner	172 (75.6)	1871 (65.2)	0.084^c^
Other^e^/Unknown	87 (24.4)	1207 (34.8)	
Region of residence			
Northeast	29 (4.4)	497 (20.5)	**<0.0001** ^c^
Midwest	117 (22.8)	603 (12.5)	
South	25 (31.3)	326 (10.2)	
West	88 (41.5)	1652 (56.8)	
Year of diagnosis			
1991	82 (5.5)	664 (4.3)	**0.001** ^b^
1995	68 (5.4)	960 (6.9)	
2000	38 (9.7)	432 (11.6)	
2005	14 (13.2)	381 (24.1)	
2010	24 (16.4)	295 (27.5)	
2015	33 (49.8)	346 (25.6)	
Insurance status			
Insured (Private, Medicare, Other^f^)	230 (92.7)	2677 (88.8)	0.072^c^
Any Medicaid^g^	21 (6.2)	266 (7.7)	
Uninsured/Unknown	8 (1.1)	135 (3.5)	
Charlson comorbidity score			
0	219 (79.7)	2604 (80.8)	0.772^c^
≥1	40 (20.3)	474 (19.2)	
Tumor size, cm			
<2.0	164 (63.6)	1845 (61.0)	0.840^c^
2.0–4.0	15 (13.8)	403 (17.4)	
>4.0	8 (8.8)	198 (7.9)	
Unknown	72 (13.8)	632 (13.7)	
Tumor grade			
Well/Moderately differentiated	75 (55.5)	1096 (46.7)	0.074^c^
Poorly differentiated/Undifferentiated	57 (29.5)	794 (41.6)	
Unknown	127 (15.0)	1188 (11.7)	
Margin status			
Positive	218 (3.7)	2605 (5.7)	0.385^c^
Negative	16 (94.7)	311 (91.6)	
Unknown	25 (1.6)	162 (2.7)	
ER status			
Positive	91 (64.9)	972 (58.7)	0.518^c^
Negative	19 (12.2)	213 (11.7)	
Test not done/Unknown	149 (22.9)	1893 (29.6)	
Laterality			
Right	116 (52.1)	1486 (49.6)	0.645^c^
Left	143 (47.9)	1592 (50.4)	
Locoregional treatment			
BCS only	70 (41.6)	790 (24.7)	**0.002** ^c^
BCS + PORT	81 (36.9)	1185 (47.6)	
Mastectomy^h^	108 (21.5)	1103 (26.7)	
Time to surgery, days			
<30	200 (54.0)	2238 (59.7)	**0.049** ^b^
31–60	46 (25.1)	544 (27.0)	
>60	13 (20.9)	296 (13.3)	
Hospital bed size			
<200^i^	131 (56.2)	663 (24.5)	**0.001** ^b^
200–499	102 (30.2)	1694 (53.1)	
≥500	26 (13.6)	721 (22.4)	
Hospital classification			
Public	82 (32.1)	360 (14.7)	**<0.0001** ^c^
Private	177 (67.9)	2718 (85.3)	
Approved residency program			
Yes	81 (24.3)	1866 (53.3)	**<0.0001** ^c^
No/unknown	178 (75.7)	1212 (46.7)	

*Note*. Bold values indicate significance at *α* = 0.05.

Abbreviations: BCS, breast‐conserving surgery; CM, centimeter; DCIS, ductal carcinoma in situ; ER, estrogen‐receptor; PORT, post‐operative radiation therapy.

^a^ Percentages based on sample weights.

^b^ *p*‐values based on weighted logistic regression for ordinal variables.

^c^ *p*‐values based on weighted Rao‐Scott chi‐square test for categorical variables.

^d^ Includes American Indians, Alaskan Natives, Asians, Hispanics, and Pacific Islanders.

^e^ Includes divorced, separated, single, and widowed.

^f^ Includes CHAMPUS, Veterans Affairs, and other governmental insurance.

^g^ Includes Indian Health Service.

^h^ Mastectomy only and mastectomy with PORT.

^i^ Includes outpatient facilities and physician offices.

### 5.1 Cancer‐directed surgery

**FIGURE 1:**
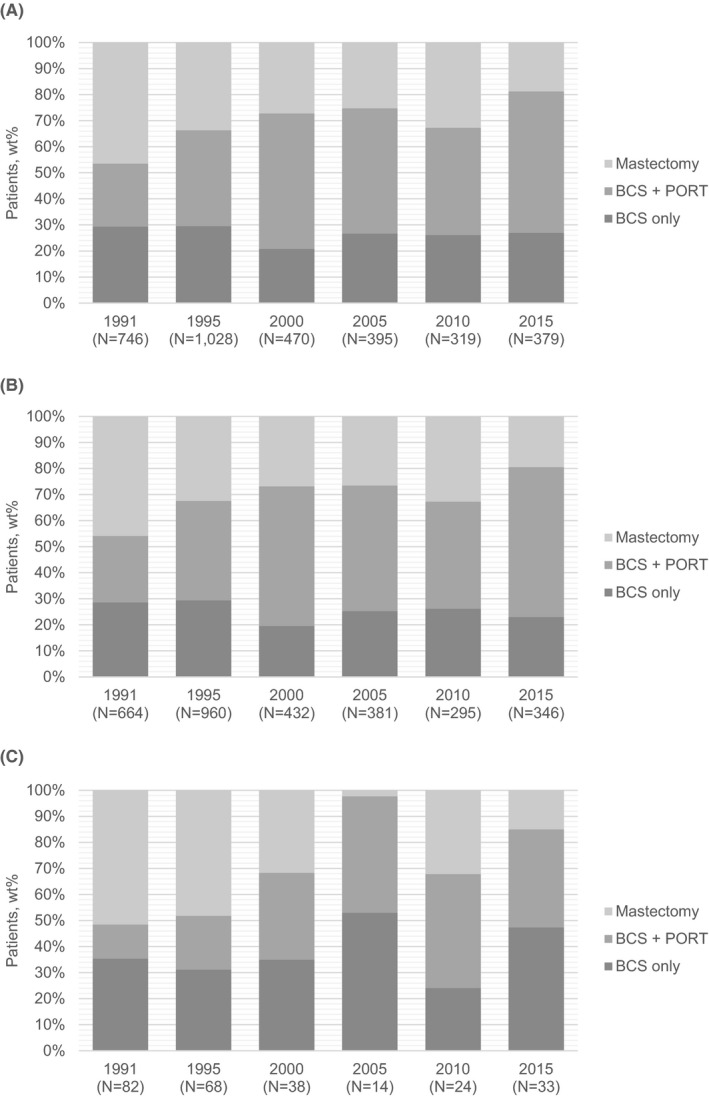
Locoregional treatment trends among ductal carcinoma in situ women by year of diagnosis. (A) All. (B) Urban. (C) Rural. BCS, breast‐conserving surgery; PORT, post‐operative radiation therapy; wt%, weighted percentage

After adjustment, there was no overall difference between rural and urban patients in the surgical approach (aOR = 0.65; 95% CI 0.37–1.14) (Table S1). However, in exploratory effect modification analyses, a significant interaction was found between rural–urban residence and diagnosis year (Table 2). In 1991/1995, rural patients were almost twice as likely to undergo mastectomy compared to urban patients (aOR = 1.78; 95% CI 1.09–2.91). In contrast, rural patients were not more likely to undergo mastectomy than urban patients in more recent diagnosis years (2000/2005 aOR = 0.57 [95% CI 0.21–1.55]; 2010/2015 aOR = 0.53 [95% CI 0.23–1.18]). The odds of mastectomy were greater in 1991/1995 compared with 2010/2015 for both rural (aOR = 4.95 [95% CI 2.32–10.6]) and urban (aOR = 2.78 [95% CI 1.56–4.96]) patients. No multiplicative effect modification was observed between rural–urban residence and region of diagnosis. Figure 1 shows the weighted percentage of patients receiving specific treatments over time. Overall, the proportion of all patients undergoing BCS with PORT increased by 30% (from 24% to 54%) between 1991 and 2015 (Figure 1A). In contrast, there was an overall decline in the rates of mastectomy (from 47% to 19%) and BCS alone (from 29% to 27%). Similar patterns were observed among urban patients over time with a 32% increase in the use of PORT following BCS and a 27% decline in the use of mastectomy. Rates of BCS alone remained relatively stable (from 29% to 23%) (Figure 1B). Unlike urban patients, the proportion of rural patients (Figure 1C) undergoing BCS with PORT increased by only 25% (from 13% to 38%) between 1991 and 2015 with a 12% increase in the rate of those undergoing BCS alone (from 35% to 47%).

**TABLE 2:** Modification of the effect of rurality on surgery choice for DCIS by year of diagnosis

	Urban		Rural		ORs (95% CI) for rural–urban status within strata of year of diagnosis
	*N* with mastectomy/with BCS	OR (95% CI)	*N* with mastectomy/with BCS	OR (95% CI)	
1991/1995	633/991	**2.78 (1.56–4.96); *p* = 0.001**	74/76	**4.95 (2.32–10.6); *p* < 0.0001**	**1.78 (1.09–2.91); *p* = 0.022**
2000/2005	267/546	1.11 (0.69–1.78); *p* = 0.679	15/37	0.63 (0.21–1.85); *p* = 0.398	0.57 (0.21–1.55); *p* = 0.268
2010/2015	203/438	1.00	19/38	0.52 (0.23–1.18); *p* = 0.117	0.53 (0.23–1.18); *p* = 0.117

*Notes*. Bold values indicate significance at *α* = 0.05.

ORs are adjusted for age, race/ethnicity, region of residence, insurance status, Charlson comorbidity score, tumor size, tumor grade, time to surgery, hospital bed size, hospital classification, and hospital residency program.

Abbreviations: BCS, breast‐conserving surgery; CI, confidence interval; DCIS, ductal carcinoma in situ; OR, odds ratio.

In univariate analyses, the odds of mastectomy were significantly lower among women aged 50 years or older and among those surgically treated at facilities with fewer than 200 beds. The odds of mastectomy were greater among those residing in areas of the Midwest or South, those diagnosed in 1991/1995, those diagnosed with tumors equal to or greater than 2.0 centimeters in size or of unknown size, and those diagnosed with poorly differentiated/undifferentiated tumors or tumors of unknown grade. Additionally, the odds of mastectomy increased with the number of days between initial biopsy and cancer‐directed surgery.

### 5.2 Post‐operative radiation therapy with breast‐conserving surgery

**FIGURE 2:**
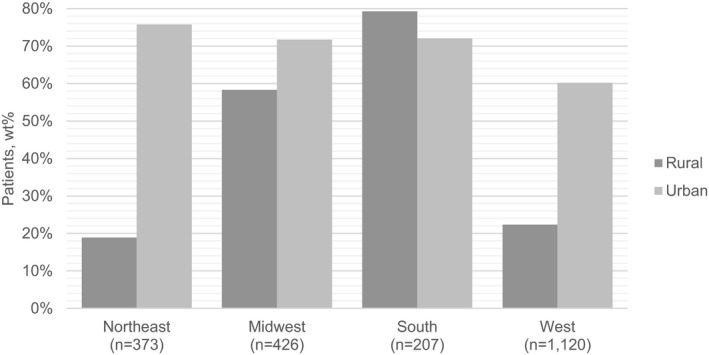
Use of post‐operative radiation therapy following breast‐conserving surgery among ductal carcinoma in situ women by region and rural–urban residence. Wt%, weighted percentage

Rural–urban residence was significantly associated with use of PORT in which rural patients were less likely to receive PORT following BCS compared to urban patients (aOR = 0.35; 95% CI 0.18–0.67) (Table S2). In exploratory effect modification analyses, we observed a significant interaction between rural–urban residence and region (Table 3). Rural patients were particularly less likely than urban patients to receive PORT in the Northeast (aOR = 0.07; 95% CI 0.01–0.41) and West (aOR = 0.19; 95% CI 0.08–0.49). These differences were not observed in areas of the Midwest (aOR = 0.55; 95% CI 0.08–3.90) or South (aOR = 1.08; 95% CI 0.38–3.08). Weighted percentages of rural and urban patients treated with BCS and PORT for each geographic region are presented in Figure 2. No multiplicative effect modification was observed between rural–urban residence and year of diagnosis. A subgroup analysis examining factors associated with PORT use among mastectomy patients was not performed due to the small sample size, which would have resulted in imprecise risk estimates.

**TABLE 3:** Modification of the effect of rurality on PORT following BCS for DCIS by region of residence

	Urban		Rural		ORs (95% CI) for rural–urban status within strata of region
	*N* with PORT/without PORT	OR (95% CI)	*N* with PORT/without PORT	OR (95% CI)	
Northeast	249/103	**2.46 (1.26–4.80); *p* = 0.008**	9/12	**0.17 (0.03–0.99); *p* = 0.049**	**0.07 (0.01–0.41); *p* = 0.004**
Midwest	237/133	1.97 (1.00–3.88); *p* = 0.051	33/23	1.08 (0.13–8.70); *p* = 0.943	0.55 (0.08–3.90); *p* = 0.549
South	122/69	1.47 (0.69–2.99); *p* = 0.332	14/2	1.56 (0.62–3.94); *p* = 0.349	1.08 (0.38–3.08); *p* = 0.881
West	577/485	1.00	25/33	**0.19 (0.08–0.49); *p* = 0.001**	**0.19 (0.08–0.49); *p* = 0.001**

*Notes*. Bold values indicate significance at *α* = 0.05.

ORs are adjusted for age at diagnosis, race/ethnicity, year of diagnosis, insurance status, Charlson comorbidity score, tumor size, tumor grade, ER status, margin status, hospital bed size, hospital classification, and hospital residency program.

Abbreviations: BCS, breast‐conserving surgery; CI, confidence interval; DCIS, ductal carcinoma in situ; ER, estrogen‐receptor; OR, odds ratio; PORT, post‐operative radiation therapy.

In univariate analyses, the odds of receiving PORT following BCS were lower among women aged 70 years or older, those diagnosed in 1991/1995, and those with an unknown estrogen‐receptor status. The odds of PORT with BCS were significantly greater among patients residing in areas of the Northeast or South and among those diagnosed with poorly differentiated/undifferentiated tumors. In addition, the odds of PORT increased with increasing tumor size.

## 6 DISCUSSION

Rural patients diagnosed with DCIS were less likely than urban patients to receive PORT following BCS in our analysis and, during 1991/1995 only, significantly more likely to receive mastectomy than BCS. While previous research has identified geographic and rural–urban differences in treatment for patients diagnosed with early‐stage, invasive breast cancer,^14^, ^15^, ^16^, ^17^, ^18^, ^19^ this is the first large, population‐based study to characterize a recent 25‐year trend in locoregional treatment and evaluate differences between rural and urban DCIS patients. Previous studies evaluating rural–urban treatment differences have been sparse and limited by less recent time periods and smaller geographic regions.^20^, ^21^, ^22^

Consistent with previous observations,^11^, ^12^ rates of PORT following BCS among all patients within our study population increased gradually throughout the study period. However, the rates of specific treatments differed between rural and urban patients over time. Among patients residing in urban areas, the use of PORT following BCS increased by 33% between 1991 and 2015 while the rates of BCS only remained relatively stable. Conversely, we observed a smaller increase (25%) in the rate of PORT following BCS among rural patients as well as a 12% increase in the rate of BCS only, suggestive of a continued rural disparity in access to PORT.

In our multivariable analyses, we adjusted for several clinical and non‐clinical factors that contributed to variation in the surgical management of DCIS. We observed a meaningful interaction between rural–urban residence and year of diagnosis. Both rural and urban patients diagnosed in 1991/1995 were significantly more likely to undergo mastectomy compared to urban patients diagnosed in 2010/2015. Rural residence was associated with a significantly greater odds of mastectomy compared with urban residence during 1991/1995, but not thereafter. During the 1980s, treatment of early‐stage invasive breast cancer began to shift away from mastectomy and towards BCS as clinical trial data emerged suggesting similar rates of survival, especially when BCS was followed by PORT.^28^ The acceptance of BCS with PORT as appropriate therapy for stage I or II breast cancer resulted in an increased interest in the use of BCS for the management of DCIS.^29^ However, given the absence of randomized controlled trial data directly comparing mastectomy to BCS for DCIS, the adoption rate of BCS in the 1990s was not uniform across all geographic areas which may reflect differences in patient or physician preferences or availability of radiotherapy facilities.^30^, ^31^, ^32^, ^33^ Using data collected by the Louisiana Tumor Registry between 1988 and 1999, Wu et al.^33^ found that DCIS patients residing in rural areas were less likely to receive lumpectomy compared to urban residents. However, consistent with our findings, the differences were only significant during the first two study periods, 1988–1991 and 1992–1996, suggesting that the differences between rural and urban patients in the use of BCS decreased over time.^33^

In terms of patient, tumor, and hospital characteristics, the odds of mastectomy use were greater among patients under the age of 50 at the time of diagnosis, among those residing in the Midwest or South, and among patients diagnosed with high‐risk tumors (defined by tumor size and grade). Such age‐related and geographic differences have been previously documented among women undergoing treatment for DCIS.^11^, ^12^, ^30^, ^31^, ^32^ A study by Shiyanbola et al.^11^ using data from the National Cancer Data Base (NCDB) between 1998 and 2011 found that DCIS patients younger than 45 and those residing in areas outside of the Northeast were significantly more likely to receive mastectomy. In addition, mastectomy use was significantly associated with quality‐of‐care metrics including longer time between biopsy and surgery and large hospital bed size (≥500 beds). Among patients treated at a single academic medical institution in the Northeast between 2000 and 2003, Sue et al.^34^ reported that the average time to treatment was 20 days longer among patients undergoing mastectomy compared to those electing for treatment with BCS, though the reasons for this delay were unclear and appear unrelated to the use of immediate postmastectomy reconstruction. Similar findings have been previously documented among patients with nonmetastatic, invasive breast cancer.^35^

Currently, BCS with PORT is considered the standard of care in the management of DCIS.^5^ Based on results from several randomized clinical trials, radiation has been found to be beneficial in preventing the recurrence of localized breast cancer compared to lumpectomy alone,^36^, ^37^, ^38^, ^39^, ^40^, ^41^ with similar survival to that of mastectomy.^42^, ^43^ Given the risk of morbidity associated with radiation use, recent efforts have been made to identify a select group of patients thought to be low‐risk for recurrence in which radiation therapy may be safely omitted including patients diagnosed with small areas of low‐grade DCIS.^44^, ^45^, ^46^, ^47^, ^48^

Among patients undergoing BCS, 56% of rural and 29% of urban patients did not receive PORT in 2015, the most recent year of POC data available. Rural DCIS patients had a significantly lower odds of receiving PORT following BCS which is consistent with findings of DCIS patients^20^, ^33^ and early‐stage breast cancer patients.^49^, ^50^ Recent studies of invasive breast cancer suggest differences in PORT use by rural–urban residence may be driven by accessibility to radiation services including increased travel distance to the nearest radiation facility.^49^, ^50^

Our description of geographic differences in the use of PORT are consistent with other studies.^9^, ^11^, ^12^ We extended previous research by examining differences in PORT use for DCIS patients by rural–urban residence within geographic regions. In exploratory effect modification analyses, rural patients residing in the Northeast and West were significantly less likely to receive PORT following BCS compared to their urban counterparts. Such variation may be attributable to differences in patient‐provider preferences or barriers to accessing specific treatments including increased daily travel distances to radiation facilities for the duration of several weeks. Further research is needed to identify challenges to receiving PORT specific to rural patients residing in separate geographic areas. These findings may guide targeted interventions aimed at improving access to radiation therapy.

Consistent with previous studies,^9^, ^10^ we found an increased likelihood of PORT use following BCS over time and among younger patients diagnosed with tumors considered to be high‐risk for local recurrence suggesting physicians are increasingly following NCCN practice guidelines.^5^

Our study has limitations that warrant consideration. Rurality was dichotomized into “rural” and “urban” based on RUCC codes assigned to each patient's county of residence at the time of diagnosis, which may have prevented the ability to observe differences between rural and urban patients residing within the same county. Another potential limitation is the small sample size of rural patients that may have reduced statistical power and led to imprecise estimates. Nevertheless, we found important differences between rural and urban patients in the use of PORT following BCS. Additionally, PORT was based solely on the sequence of radiation with surgery. The extent of PORT, including the dose and number of treatments, was not available in the POC data and therefore could not be assessed. Finally, while the SEER POC data do include information beyond what is routinely collected by SEER registries such as hospital characteristics (i.e., bed size, classification, residency training program), information about treatment decision making including patient preferences or physician recommendations were not available.

## 7 CONCLUSION

In summary, locoregional treatments for DCIS have evolved substantially between 1991 and 2015 in the United States. We observed an overall increase in the use of BCS compared to mastectomy, coinciding with the introduction of clinical practice guidelines. However, despite evidence that suggests PORT reduces the risk of local recurrence, omission of PORT following BCS remains high among rural patients. Further research is needed to examine the impact of rural residence on treatment choices and develop targeted interventions to increase rates of PORT with the goal of ensuring equitable care among all breast cancer patients.

## CONFLICT OF INTEREST

The authors made no disclosures.

## AUTHOR CONTRIBUTIONS

Danielle Riley contributed to conceptualization, methodology, formal analysis, writing‐original draft, and writing‐review and editing. Elizabeth A. Chrischilles contributed to supervision, writing‐review and editing. Ingrid M. Lizarraga contributed to writing‐review and editing. Mary Charlton contributed to writing‐review and editing. Brian J. Smith contributed to methodology, formal analysis, writing‐review and editing. Charles F. Lynch contributed to conceptualization, supervision, writing‐review and editing.

## Supporting information


Table S1‐S2

## Data Availability

Access to the Patterns of Care dataset can be requested here: https://healthcaredelivery.cancer.gov/poc/access.html
