# Supplementary material for: Rural–urban differences in secular trends of locoregional treatment for ductal carcinoma in situ: A patterns of care analysis
Source: Cancer Med. 2022 Feb 11;11(11):2284–95. doi: 10.1002/cam4.4605 (PMC9160801; doi:10.1002/cam4.4605)
Supplement: Supplementary file 1 — Table S1‐S2 [file CAM4-11-2284-s001.docx]

| **Table S1.** Odds of receiving mastectomy among DCIS patients | | | | | |
| --- | --- | --- | --- | --- | --- |
|  | Mastectomy | BCS |  |  |  |
|  | (n=1,211) | (n=2,126) | Unadjusted | Adjusted | Adjusted |
|  | N (wt%^a^) | N (wt%^a^) | OR (95% CI) | *P*-value | OR^b^ (95% CI) |
| **Rural-urban residence** |  |  |  | 0.132 |  |
| Urban | 1,103 (92.7) | 1,975 (90.1) | 1.00 Referent |  | 1.00 Referent |
| Rural | 108 (7.3) | 151 (9.9) | 0.72 (0.46-1.11) |  | 0.65 (0.37-1.14) |
| **Age at diagnosis, years** |  |  |  | **0.0002** |  |
| <50 | 549 (37.4) | 829 (20.8) | 1.00 Referent |  | 1.00 Referent |
| 50-59 | 276 (28.0) | 515 (26.5) | **0.59 (0.37-0.92)** |  | 0.64 (0.41-1.00) |
| 60-69 | 215 (20.9) | 432 (25.9) | **0.45 (0.30-0.68)** |  | **0.44 (0.29-0.68)** |
| ≥70 | 171 (13.7) | 350 (26.8) | **0.28 (0.17-0.48)** |  | **0.35 (0.20-0.64)** |
| **Race/Ethnicity** |  |  |  | 0.085 |  |
| Non-Hispanic, White | 663 (65.7) | 1,116 (70.2) | 1.00 Referent |  | 1.00 Referent |
| Non-Hispanic, Black | 231 (10.5) | 429 (9.7) | 1.16 (0.79-1.70) |  | 0.96 (0.62-1.51) |
| Other^c^/Unknown | 317 (23.8) | 581 (20.1) | 1.27 (0.90-1.78) |  | **1.55 (1.01-2.37)** |
| **Region of residence** |  |  |  | **0.009** |  |
| Northeast | 153 (16.4) | 373 (20.0) | 0.97 (0.59-1.57) |  | 1.23 (0.71-2.14) |
| Midwest | 294 (18.8) | 426 (11.4) | **1.93 (1.24-3.02)** |  | **2.41 (1.32-4.42)** |
| South | 144 (15.8) | 207 (10.8) | **1.73 (1.09-2.74)** |  | **2.32 (1.24-4.36)** |
| West | 620 (49.0) | 1,120 (57.8) | 1.00 Referent |  | 1.00 Referent |
| **Year of diagnosis** |  |  |  | **<0.0001** |  |
| 1991/1995 | 707 (16.0) | 1,067 (9.4) | **1.84 (1.36-2.49)** |  | **3.11 (1.76-5.47)** |
| 2000/2005 | 282 (32.9) | 583 (35.1) | 1.02 (0.70-1.48) |  | 1.12 (0.70-1.78) |
| 2010/2015 | 222 (51.1) | 476 (55.5) | 1.00 Referent |  | 1.00 Referent |
| **Insurance status** |  |  |  | **0.032** |  |
| Insured (Private, Medicare, Other^d^) | 1,056 (90.1) | 1,851 (88.8) | 1.00 Referent |  | 1.00 Referent |
| Any Medicaid^e^ | 104 (7.6) | 183 (7.6) | 0.99 (0.60-1.62) |  | 0.70 (0.36-1.35) |
| Uninsured/Unknown | 51 (2.3) | 92 (3.6) | 0.64 (0.33-1.23) |  | **0.44 (0.23-0.85)** |
| **Charlson comorbidity score** |  |  |  | 0.997 |  |
| 0 | 1,030 (83.0) | 1,793 (79.8) | 1.00 Referent |  | 1.00 Referent |
| ≥1 | 181 (17.0) | 333 (20.2) | 0.81 (0.53-1.23) |  | 1.00 (0.63-1.58) |
| **Tumor size, cm** |  |  |  | **<0.0001** |  |
| <2.0 | 609 (47.9) | 1,400 (66.2) | 1.00 Referent |  | 1.00 Referent |
| 2.0-4.0 | 179 (21.2) | 239 (15.6) | **1.86 (1.15-3.02)** |  | **2.26 (1.37-3.72)** |
| >4.0 | 125 (12.5) | 81 (6.3) | **2.77 (1.54-4.99)** |  | **2.90 (1.60-5.25)** |
| Unknown | 298 (18.4) | 406 (11.9) | **2.12 (1.33-3.38)** |  | **2.25 (1.37-3.70)** |
| **Tumor grade** |  |  |  | **0.035** |  |
| Well/Moderately differentiated | 345 (36.2) | 826 (51.7) | 1.00 Referent |  | 1.00 Referent |
| Poorly differentiated/Undifferentiated | 348 (49.3) | 503 (37.2) | **1.89 (1.27-2.82)** |  | **1.73 (1.14-2.63)** |
| Unknown | 518 (14.5) | 797 (11.1) | **1.87 (1.28-2.73)** |  | 1.24 (0.69-2.23) |

| **Table S1.** Odds of receiving mastectomy among DCIS patients (*Continued*) | | | | | |
| --- | --- | --- | --- | --- | --- |
| **Time to surgery, days** |  |  |  | **<0.0001** |  |
| <30 | 786 (45.8) | 1,652 (64.1) | 1.00 Referent |  | 1.00 Referent |
| 31-60 | 255 (30.8) | 335 (25.4) | **1.70 (1.11-2.60)** |  | **1.93 (1.23-3.03)** |
| >60 | 170 (23.4) | 139 (10.5) | **3.12 (1.94-5.00)** |  | **4.07 (2.43-6.79)** |
| **Hospital bed size** |  |  |  | **0.032** |  |
| <200^f^ | 273 (18.6) | 521 (30.7) | **0.44 (0.27-0.73)** |  | **0.55 (0.31-0.97)** |
| 200-499 | 668 (54.5) | 1,128 (49.6) | 0.80 (0.51-1.26) |  | 0.87 (0.51-1.47) |
| ≥500 | 270 (26.9) | 477 (19.7) | 1.00 Referent |  | 1.00 Referent |
| **Hospital classification** |  |  |  | 0.949 |  |
| Public | 170 (15.8) | 272 (16.5) | 0.95 (0.55-1.63) |  | 1.02 (0.53-1.99) |
| Private | 1,041 (84.2) | 1,854 (83.5) | 1.00 Referent |  | 1.00 Referent |
| **Approved residency program** |  |  |  | **0.042** |  |
| Yes | 688 (52.1) | 1,259 (50.1) | 1.00 Referent |  | 1.00 Referent |
| No/Unknown | 523 (47.9) | 867 (49.9) | 0.93 (0.65-1.32) |  | **1.47 (1.01-2.14)** |
| Abbreviations: BCS=breast-conserving surgery; CI=confidence interval; CM=centimeter; DCIS=ductal carcinoma *in situ*; OR=odds ratio | | | | | |
| Bold values indicate significance at α=0.05 | | | | | |
| ^a^Percentages based on sample weights | | | | | |
| ^b^Adjusted for all other variables in the table | | | | | |
| ^c^Includes American Indians, Alaskan Natives, Asians, Hispanics and Pacific Islanders | | | | | |
| ^d^Includes CHAMPUS, Veterans Affairs, and other governmental insurance | | | | | |
| ^e^Includes Indian Health Service | | | | | |
| ^f^Includes outpatient facilities and physician offices | | | | | |

| **Table S2.** Odds of receiving PORT following BCS among DCIS patients | | | | | |
| --- | --- | --- | --- | --- | --- |
|  | PORT | No PORT |  |  |  |
|  | (n=1,342) | (n=860) | Unadjusted | Adjusted | Adjusted |
|  | N (wt%^a^) | N (wt%^a^) | OR (95% CI) | *P*-value | OR^b^ (95% CI) |
| **Rural-urban residence** |  |  |  | **0.002** |  |
| Urban | 1,185 (92.7) | 790 (85.4) | 1.00 Referent |  | 1.00 Referent |
| Rural | 81 (7.3) | 70 (14.6) | **0.46 (0.27-0.78)** |  | **0.35 (0.18-0.67)** |
| **Age at diagnosis, years** |  |  |  | **0.043** |  |
| <50 | 543 (22.6) | 286 (17.6) | 1.00 Referent |  | 1.00 Referent |
| 50-59 | 306 (26.3) | 209 (26.7) | 0.77 (0.50-1.19) |  | 0.83 (0.52-1.32) |
| 60-69 | 260 (28.5) | 172 (21.3) | 1.04 (0.65-1.66) |  | 0.83 (0.50-1.40) |
| ≥70 | 157 (22.6) | 193 (4.4) | **0.51 (0.29-0.90)** |  | **0.40 (0.22-0.76)** |
| **Race/Ethnicity** |  |  |  | **0.042** |  |
| Non-Hispanic, White | 647 (70.9) | 469 (68.8) | 1.00 Referent |  | 1.00 Referent |
| Non-Hispanic, Black | 272 (11.1) | 157 (7.2) | 1.51 (0.98-2.32) |  | 1.30 (0.80-2.10) |
| Other^c^/Unknown | 347 (18.0) | 234 (24.0) | 0.73 (0.48-1.11) |  | 0.68 (0.42-1.10) |
| **Region of residence** |  |  |  | **0.019** |  |
| Northeast | 258 (23.3) | 115 (14.2) | **2.17 (1.27-3.71)** |  | **2.43 (1.27-4.66)** |
| Midwest | 270 (12.4) | 156 (9.7) | 1.70 (0.90-3.20) |  | **2.32 (1.11-4.85)** |
| South | 136 (12.5) | 71 (7.7) | **2.14 (1.20-3.82)** |  | **2.02 (1.02-3.99)** |
| West | 602 (51.8) | 518 (68.4) | 1.00 Referent |  | 1.00 Referent |
| **Year of diagnosis** |  |  |  | 0.594 |  |
| 1991/1995 | 554 (7.6) | 513 (12.5) | **0.60 (0.42-0.85)** |  | 1.23 (0.63-2.39) |
| 2000/2005 | 391 (36.6) | 192 (32.5) | 1.11 (0.70-1.76) |  | 1.35 (0.76-2.40) |
| 2010/2015 | 321 (55.8) | 155 (55.0) | 1.00 Referent |  | 1.00 Referent |
| **Insurance status** |  |  |  | 0.632 |  |
| Insured (Private, Medicare, Other^d^) | 1,112 (87.9) | 739 (90.6) | 1.00 Referent |  | 1.00 Referent |
| Any Medicaid^e^ | 100 (7.7) | 83 (7.3) | 1.09 (0.63-1.91) |  | 0.95 (0.54-1.66) |
| Uninsured/Unknown | 54 (4.4) | 38 (2.1) | 2.14 (0.90-5.12) |  | 1.53 (0.62-3.73) |
| **Charlson comorbidity score** |  |  |  | 0.878 |  |
| 0 | 1,067 (79.8) | 726 (79.7) | 1.00 Referent |  | 1.00 Referent |
| ≥1 | 199 (20.2) | 134 (20.3) | 0.99 (0.57-1.71) |  | 1.05 (0.59-1.84) |
| **Tumor size, cm** |  |  |  | **0.001** |  |
| <2.0 | 823 (60.8) | 577 (75.7) | 1.00 Referent |  | 1.00 Referent |
| 2.0-4.0 | 162 (19.1) | 77 (9.4) | **2.54 (1.45-4.47)** |  | **2.96 (1.53-5.72)** |
| >4.0 | 60 (8.5) | 21 (2.3) | **4.57 (1.86-11.2)** |  | **4.46 (1.49-13.4)** |
| Unknown | 221 (11.6) | 185 (12.6) | 1.14 (0.65-2.02) |  | 1.12 (0.61-2.04) |
| **Tumor grade** |  |  |  | **<0.0001** |  |
| Well/Moderately differentiated | 476 (44.9) | 350 (63.6) | 1.00 Referent |  | 1.00 Referent |
| Poorly differentiated/Undifferentiated | 395 (46.5) | 108 (20.8) | **3.16 (1.90-5.24)** |  | **2.92 (1.80-4.73)** |
| Unknown | 395 (8.6) | 402 (15.6) | 0.78 (0.50-1.21) |  | 0.80 (0.43-1.49) |

| **Table S2.** Odds of receiving PORT following BCS among DCIS patients (*Continued*) | | | | | |
| --- | --- | --- | --- | --- | --- |
| **ER status** |  |  |  | 0.102 |  |
| Positive | 472 (62.7) | 219 (57.1) | 1.00 Referent |  | 1.00 Referent |
| Negative | 92 (13.4) | 26 (7.6) | 1.11 (0.59-2.07) |  | 0.95 (0.37-2.45) |
| Test not done/Unknown | 702 (23.9) | 615 (35.3) | **0.63 (0.46-0.86)** |  | **0.55 (0.32-0.96)** |
| **Margin status** |  |  |  | 0.552 |  |
| Negative | 1,059 (91.1) | 685 (92.2) | 1.00 Referent |  | 1.00 Referent |
| Positive | 139 (6.6) | 109 (5.2) | 1.29 (0.73-2.28) |  | 1.12 (0.60-2.09) |
| Unknown | 68 (2.3) | 66 (2.6) | 0.90 (0.51-1.58) |  | 1.42 (0.75-2.70) |
| **Hospital bed size** |  |  |  | 0.340 |  |
| <200^f^ | 303 (29.5) | 218 (32.8) | 0.63 (0.37-1.05) |  | 1.08 (0.53-2.17) |
| 200-499 | 650 (48.4) | 478 (51.9) | 0.65 (0.39-1.09) |  | 0.77 (0.42-1.44) |
| ≥500 | 313 (22.1) | 164 (15.3) | 1.00 Referent |  | 1.00 Referent |
| **Hospital classification** |  |  |  | 0.447 |  |
| Public | 160 (18.3) | 112 (13.2) | 1.48 (0.87-2.53) |  | 1.28 (0.67-2.45) |
| Private | 1,106 (81.7) | 748 (86.8) | 1.00 Referent |  | 1.00 Referent |
| **Approved residency program** |  |  |  | 0.684 |  |
| Yes | 785 (53.2) | 474 (44.7) | 1.00 Referent |  | 1.00 Referent |
| No/Unknown | 481 (46.8) | 386 (55.3) | 0.71 (0.48-1.06) |  | 1.10 (0.69-1.77) |
| Abbreviations: BCS=breast-conserving surgery; CI=confidence interval; CM=centimeter; DCIS=ductal carcinoma *in situ*; ER=estrogen-receptor; OR=odds ratio; PORT=post-operative radiation therapy | | | | | |
| Bold values indicate significance at α=0.05 | | | | | |
| ^a^Percentages based on sample weights | | | | | |
| ^b^Adjusted for all other variables in the table | | | | | |
| ^c^Includes American Indians, Alaskan Natives, Asians, Hispanics and Pacific Islanders | | | | | |
| ^d^Includes CHAMPUS, Veterans Affairs, and other governmental insurance | | | | | |
| ^e^Includes Indian Health Service | | | | | |
| ^f^Includes outpatient facilities and physician offices | | | | | |
